# Improved salt tolerance of *Synechococcus elongatus* PCC 7942 by heterologous synthesis of compatible solute ectoine

**DOI:** 10.3389/fmicb.2023.1123081

**Published:** 2023-02-02

**Authors:** Zhengxin Dong, Tao Sun, Weiwen Zhang, Lei Chen

**Affiliations:** ^1^Laboratory of Synthetic Microbiology, School of Chemical Engineering and Technology, Tianjin University, Tianjin, China; ^2^Frontier Science Center for Synthetic Biology and Key Laboratory of Systems Bioengineering, Ministry of Education of China, Tianjin, China; ^3^Center for Biosafety Research and Strategy, Tianjin University, Tianjin, China

**Keywords:** cyanobacteria, salt stress, compatible solutes, ectoine, transcriptome

## Abstract

Salt stress is one of the essential abiotic stresses for the survival of cyanobacteria. However, the realization of large-scale cultivation of cyanobacteria is inseparable from the utilization of abundant seawater resources. Therefore, research on the regulatory mechanism, as well as the improvement of salt tolerance of cyanobacteria is fundamental. Ectoine, a compatible solute which was found in halophilic microorganisms, has potentiality to confer salt tolerance. Here in this article, the salt tolerance of *Synechococcus elongatus* PCC 7942 (Syn7942) was significantly improved *via* expressing the ectoine biosynthetic pathway, reaching an increased final OD_750_ by 20% under 300 mM NaCl and 80% under 400 mM NaCl than that of wild-type (WT), respectively. Encouragingly, the engineered strain could even survive under 500 mM NaCl which was lethal to WT. In addition, by introducing the ectoine synthetic pathway into the sucrose-deficient strain, the salt tolerance of the obtained strain Syn7942/Δsps-ect was restored to the level of WT under 300 mM NaCl stress, demonstrating that ectoine could substitute for sucrose to combat against salt stress in Syn7942. In order to study the difference in the regulation of mechanism on the salt adaptation process after replacing sucrose with ectoine, transcriptomic analysis was performed for Syn7942/Δsps-ect and WT. The differentially expressed gene analysis successfully identified 19 up-regulated genes and 39 down-regulated genes in Syn7942/Δsps-ect compared with WT under salt stress condition. The results also showed that the global regulation of Syn7942/Δsps-ect and WT had certain differences in the process of salt adaptation, in which Syn7942/Δsps-ect reduced the demand for the intensity of sulfur metabolism in this process. This study provides a valuable reference for further salt tolerance engineering in cyanobacteria.

## Introduction

Cyanobacteria can use solar energy and CO_2_ in the air to synthesize organic compounds, realizing the negative carbon economy in the process, which have received widespread attention as autotrophic cell factories (Kato et al., 2022; Liu et al., 2022; Tan et al., 2022). As a model cyanobacterium, *Synechococcus elongatus* PCC 7942 (hereafter Syn7942), is amenable to genetic manipulation with the development of toolboxes and has been employed as a biological chassis for chemical production (Kim et al., 2017; Sun et al., 2018; Sengupta et al., 2019; Zhang M. et al., 2022). Nowadays, it is expected to carry out industrialized production of biofuels and chemicals through the large-scale cultivation of cyanobacteria (Farrokh et al., 2019; Davies et al., 2021; Wang et al., 2021), which requires the employment of seawater resources with rich reserves (Cui et al., 2020). Therefore, research on improving the salt tolerance of cyanobacteria is required to enable their cultivation in seawater resources to produce high value-added chemicals. Moreover, as a model freshwater cyanobacterium, the engineering and regulation mechanism will provide a valuable reference for further salt tolerance engineering in cyanobacteria.

Most of halophilic microorganisms accumulate organic compatible solutes to maintain the balance of osmotic pressure of the intracellular and the external environment (Gunde-Cimerman et al., 2018). Among them, ectoine was initially found in *Ectothiorhodospira halochloris* (Galinski et al., 1985) and is widely used in the fields of food, cosmetics, and medicine (Liu et al., 2021; Kauth and Trusova, 2022; Zhang H. et al., 2022). Recent studies have shown that there was also the native synthesis of ectoine in some microalgae (Fenizia et al., 2020). The biosynthetic pathway of ectoine has been analyzed, and its heterologous synthesis has been achieved in both *Escherichia coli* and *Corynebacterium glutamicum* (Giesselmann et al., 2019; Chen J. et al., 2020; Zhang H. et al., 2022). The synthesis of ectoine depends on gene *ectB* encoding L-2,4-diaminobutyrate transaminase, *ectA* encoding 2,4-diaminobutyrate acetyltransferase, *ectC* encoding ectoine synthase, and using L-aspartate-β-semialdehyde as substrate (Göller et al., 1998; Calderón et al., 2004; Schwibbert et al., 2011). However, the only compatible solute in Syn7942 is sucrose (Klähn and Hagemann, 2011). Heterologous synthesis compatible solute to improve salt tolerance has been considered a very effective method (Waditee-Sirisattha et al., 2012; Singh et al., 2013; Cui et al., 2021). For example, the heterologous synthesis of glucosylglycerol in *Synechococcus elongatus* UTEX 2973 resulted in a 62% increase in growth under 0.5 M NaCl conditions (Cui et al., 2021). Therefore, it is feasible to synthesize ectoine heterologously to increase salt tolerance in Syn7942.

In this study, aiming to engineering the salt tolerance of the model cyanobacterium Syn7942, the first study of heterologous synthesis of ectoine in cyanobacteria was achieved, and the salt tolerance of Syn7942 was successfully improved. This provides a potential solution for improving the salt tolerance of cyanobacteria while producing high value-added products. Then, the recovery of salt tolerance of the Syn7942/Δsps-ect strain was achieved by introducing the ectoine synthesis pathway into the sucrose synthesis deficient strain (Syn7942/Δsps), indicating that ectoine was able to substitute for sucrose to combat against salt stress in Syn7942. Finally, to deciphering the mechanism of improved salt tolerance in the engineered Syn7942/Δsps-ect, the transcriptional differences between strains Syn7942/Δsps-ect and WT under salt stress condition were further studied by comparative transcriptomics, and the differentially expressed genes (DEGs) were identified. This study provided valuable information for engineering salt-tolerant cyanobacteria.

## Materials and methods

### Bacterial growth conditions and salt-stress treatment

The WT and engineered Syn7942 were grown at 37°C in BG11 liquid medium (pH 7.5) or on agar plates under a light intensity of approximately 100 μmol photons m^−2^ s^−1^ in an HNY-211B Illuminating incubator Shaker of 200 rpm in 100 ml flask (Honour, Tianjin, China). Twenty-five milligrams per liter of chloramphenicol (Solarbio, Beijing, China) was added during the culture of engineering strains. Different salt concentrations of media used in the culture process were obtained by adding a suitable amount of 3 M to 0 M NaCl BG11 medium. Cell density was measured at 750 nm (OD_750_) by an ELx808 Absorbance Microplate Reader (BioTek, VT, United States). *E. coli* TOP10 was grown in LB liquid medium or LB agar plates, with 50 mg/l of chloramphenicol or spectinomycin for screening and maintaining the stability of engineered bacteria.

### Construction of strains and plasmids

The strains used in this study are listed in Table 1. Among them, *E. coli* TOP10 was used for plasmid construction. pSI-ect vector with a chloramphenicol-resistant cassette was constructed for express ectoine production cassette based on laboratory plasmid (Li et al., 2018). To knock out the sucrose production pathway and express the ectoine synthesis pathway after the knockout of the sucrose production pathway, pSPS-ect was constructed by replacing the upstream and downstream homologous arms based on pSI-ect, and pSPS was built by deleting an ectoine expression cassette based on pSPS-ect. Primers and plasmids used for this study are listed in Supplementary Table S1. All primers were synthesized by Azenta (Suzhou, China). The genes *ectA*, *ectB*, and *ectC* were synthesized by Azenta based on the *Halomonas elongata* after codon optimization. The sequences are shown in Supplementary Table S2. The template plasmids were purified by FastPure Plasmid Mini Kit (Vazyme Biotech, Nanjing, China). The target fragments were amplified by Phanta Super-Fidelity DNA Polymerase (Vazyme Biotech, Nanjing, China) and purified by FastPure Gel DNA Extraction Mini Kit (Vazyme Biotech, Nanjing, China). Then, fragments were ligated by ClonExpress MultiS One Step Cloning Kit (Vazyme Biotech, Nanjing, China) or Golden Gate (Thermo Fisher Scientific Inc., CA, United States). All constructs were verified by PCR and Sanger sequencing. After the constructed plasmids were extracted and cut through NdeI (Thermo Fisher Scientific Inc., CA, United States), and were transformed into Syn7942 by the method of natural transformation.

**Table 1:** Strains used in this study.

**Strains**	**Genotype or relevant features**	**References**
WT	*Synechococcus elongatus* PCC 7942	Laboratory storage
Syn7942/NSI-ect	NSI:P_cpc560_-ectABC-T_rbcL_; cm^R^	In this study
Syn7942/Δsps	Synpcc7942_0808:Pcat-cm^R^-T_rrnB_;cmR	In this study
Syn7942/Δsps-ect	Synpcc7942_0808:P_cpc560_-ectABC-T_rbcL_	In this study

### Ectoine extraction and measurement

One milligram liter of cultures on the 7th day was taken out and placed at −80°C. When preparing the samples, the cultures were taken out from −80°C, placed at 70°C for 2 h, centrifuged at 13,000 rpm. And the supernatant was taken for ectoine concentration determination. Based on the previous study (Ning et al., 2016), the ectoine was detected using high-performance liquid chromatography (Agilent 1,260 Series HPLC, Agilent Technologies, Santa Clara, CA, United States), and the concentration of ectoine in the samples was determined using the ectoine standard (Sigma-Aldrich, Shanghai, China). The chromatographic column was Ultimate AQ-C18, 5 μm 4.6*250 mm (Welch, China), and the mobile phase condition was ultrapure water of 0.6 ml/min. The ectoine was monitored using a UV detector at a wavelength of 210 nm.

### Transcriptomic analysis

WT and Syn7942/Δsps-ect were cultured in BG11 medium with 0 mM as control-1 and control-2 and 300 mM NaCl as experimental-1 and experimental-2, respectively. On the fourth day, samples were collected and sent to Azenta for transcriptome sequencing and data analysis. Each sample had three biological replicates. The log_2_(fold-change) > 1.5 and the *value of p* <0.05 were set as the threshold for DEGs identification. KEGG enrichment analysis was done using TBtools (Version v1.098769; Chen C. et al., 2020). There was still a small number of reads for the *Synpcc7942_0808* gene in Syn7942/Δsps-ect. In order to exclude data interference, the number of reads for the *Synpcc7942_0808* gene in Syn7942/Δsps-ect was ignored.

The transcriptome data has been uploaded to GEO database (GSE222067).

## Results and discussion

### Improved salt tolerance of Syn7942 by heterologous expression of the ectoine biosynthetic pathway

**Figure 1:**
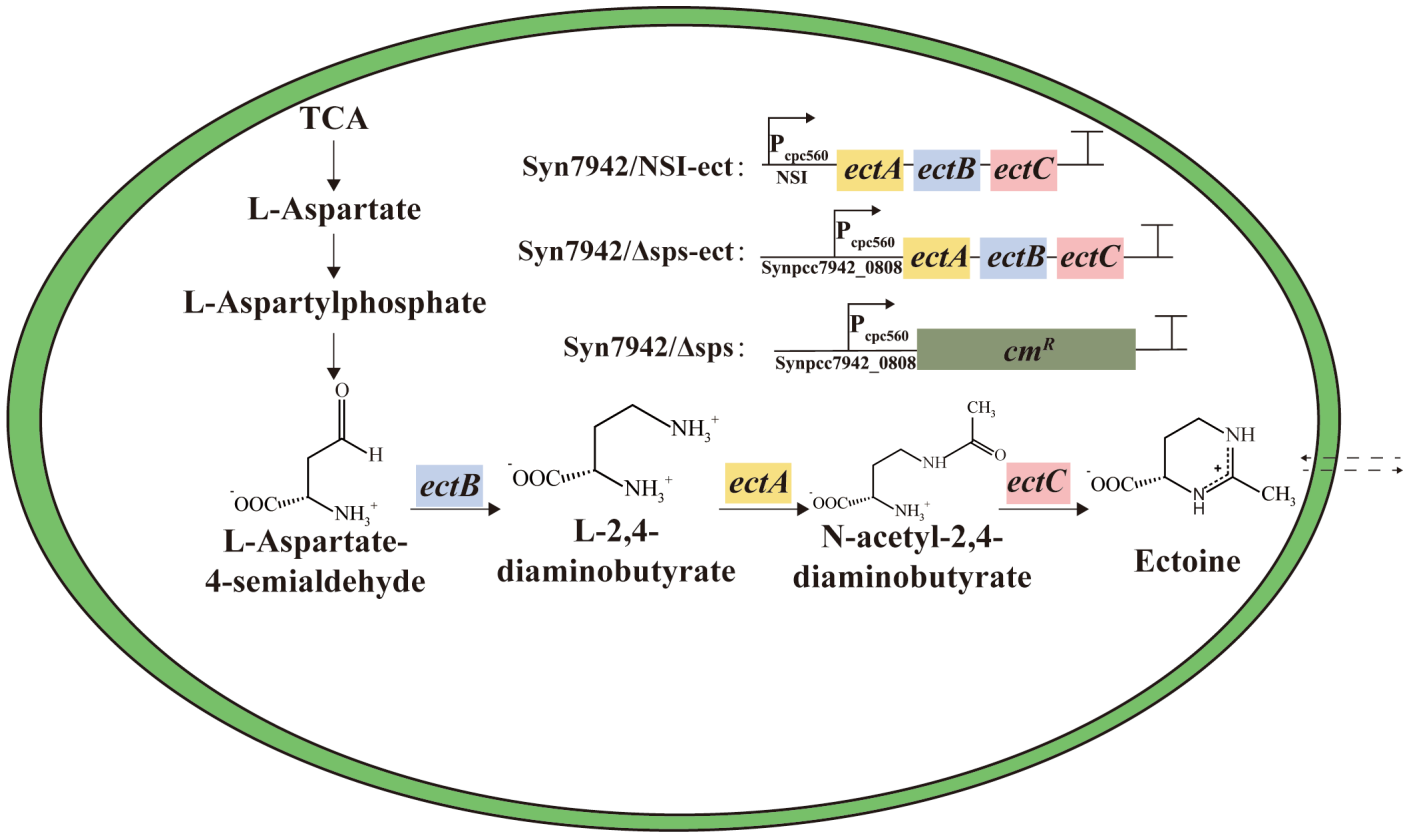
The reconstructed ectoine biosynthetic pathway in Syn7942. Syn7942/NSI-ect: indicates that the expression cassette indicated in the legend was inserted at the neutral site I (NSI); Syn7942/Δsps and Syn7942/Δsps-ect: indicate that the endogenous *Synpcc7942_0808* gene was replaced using the expression cassette in the legend; TCA: indicates tricarboxylic acid cycle.

**Figure 2:**
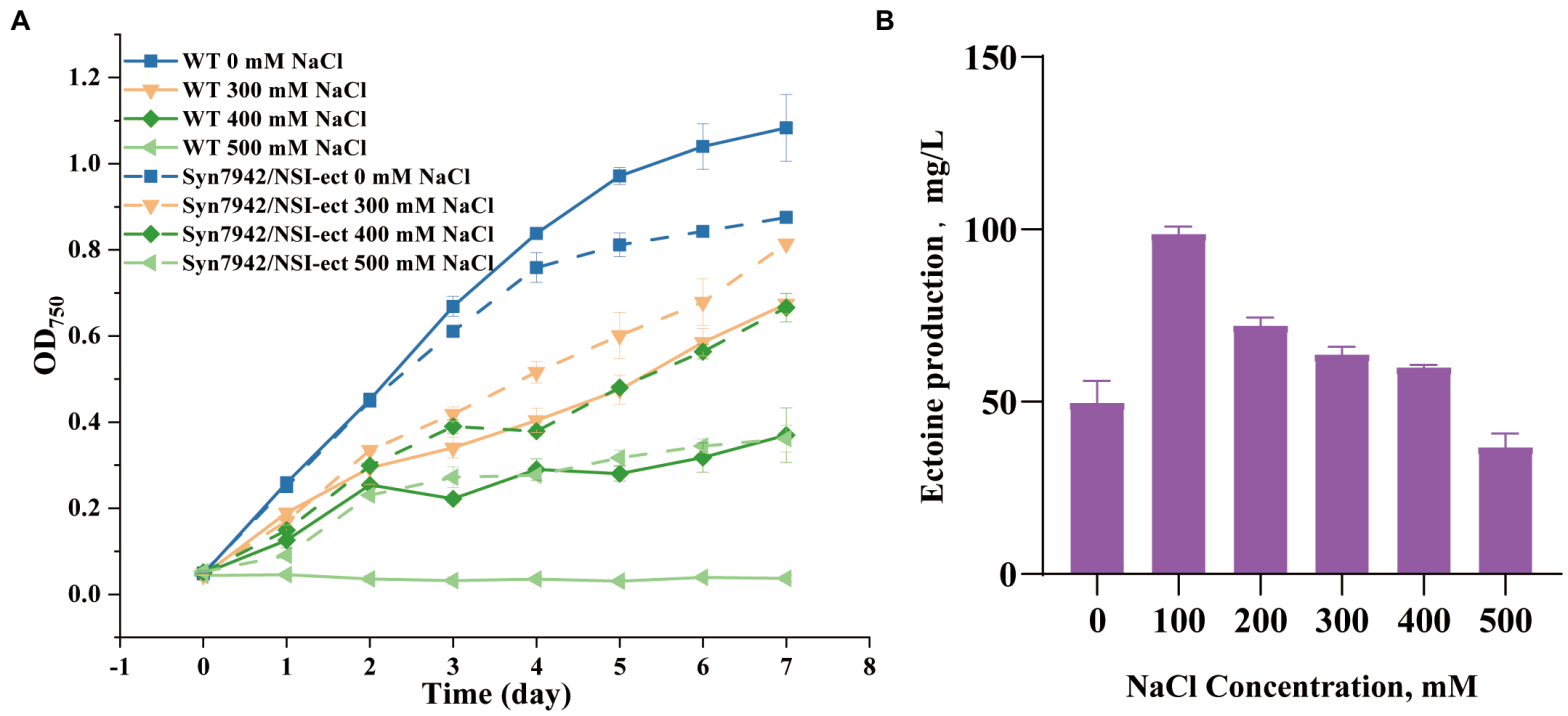
Growth curves and ectoine production of WT and Syn7942/NSI-ect. **(A)** Growth curves of WT and Syn7942/NSI-ect under 0, 300, 400, 500 mM NaCl; **(B)** Ectoine production of Syn7942/NSI-ect under 0, 100, 200, 300, 400, 500 mM NaCl.

The gene cluster *ectABC* was codon-optimized for Syn7942 and then has been chemically synthesized and introduced into the NSI site of Syn7942 afterwards. The expression is controlled by the strong promoter P_cpc560_ (Figure 1). This engineered strain was named Syn7942/NSI-ect. To evaluate the salt tolerance of Syn7942/NSI-ect, WT was selected as the control strain, and the growth curves of Syn7942/NSI-ect and WT were measured in BG11 medium with addition of 0, 100, 200, 300, 400, and 500 mM NaCl, respectively. Under the growth condition of 0 mM NaCl, the OD_750_ of Syn7942/NSI-ect was decreased by 19% compared with that of WT on the 7th day, which may be caused by the competition between ectoine synthesis pathway and endogenous amino acid metabolism pathway for L-aspartate-4-semialdehyde. And the growth of Syn7942/NSI-ect and WT was almost the same under 100 and 200 mM NaCl conditions, while the OD_750_ of Syn7942/NSI-ect on the 7th day was increased by 20% than that of WT under 300 mM NaCl and increased by 80% under the condition of 400 mM NaCl (Figure 2A; Supplementary Figures S1A,B), demonstrating the improved salt tolerance of strain Syn7942/NSI-ect. Under 500 mM NaCl condition, the OD_750_ of Syn7942/NSI-ect on the 7th day could reach 0.362 while WT could not survive at all. This suggested that the salt tolerance of Syn7942 could be improved by the introduction of ectoine biosynthesis pathway.

The production of ectoine in Syn7942/NSI-ect under different salt concentrations on the 7th day was then determined using the external standard method. The standard curve was shown in Supplementary Figure S2. As shown in Figure 2B, under the condition of 0 mM NaCl, the yield of ectoine was 49.8 mg/l in Syn7942/NSI-ect. Moreover, under 100 mM NaCl, the yield of ectoine was significantly increased, reaching 98.9 mg/l. It has also been reported that ectoine production increased with the concentration of NaCl in the environment (Ning et al., 2016; Yu et al., 2022), and this phenomenon also occurred in the synthesis of glucosylglycerol and sucrose (Song et al., 2016; Cui et al., 2021). With the further increase of salt concentration, the yield of ectoine decreased compared with under 100 mM NaCl, most probably due to the worse growth caused by salt stress.

In previous studies, the salt environment was reported to be favorable for the synthesis of ectoine (Ning et al., 2016). Here, our results showed that the synthesis of ectoine could significantly improve the salt tolerance of Syn7942/NSI-ect, and the amount of ectoine synthesis was responsive to the high-salt environment.

### Ectoine could substitute for sucrose to combat against salt stress in Syn7942

**Figure 3:**
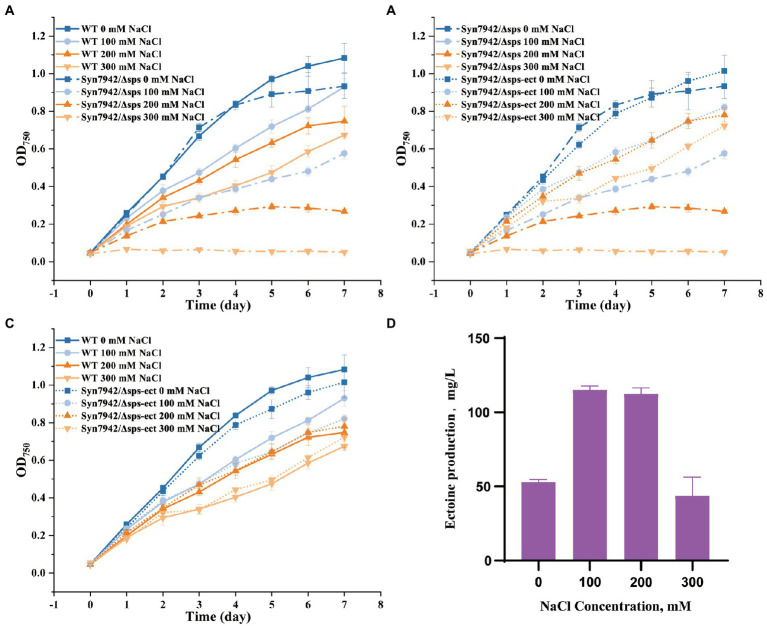
Growth curves and ectoine production of WT, Syn7942/Δsps, and Syn7942/Δsps-ect. **(A)** Growth curves of WT and Syn7942/Δsps under 0, 100, 200, 300 mM NaCl; **(B)** Growth curves of Syn7942/Δsps and Syn7942/Δsps-ect under 0, 100, 200, 300 mM NaCl; **(C)** Growth curves of WT and Syn7942/Δsps-ect under 0, 100, 200, 300 mM NaCl; **(D)** Ectoine production of Syn7942/Δsps-ect under 0, 100, 200, 300 mM NaCl.

The accumulation of compatible solutes is an essential way for microorganisms to resist the high-salt environment, and the only compatible solute in Syn7942 is sucrose (Klähn and Hagemann, 2011). The loss of the sucrose synthesis pathway led to the generation of a salt-sensitive strain, which indicated that sucrose was the only compatible solute. However, sucrose is not considered an ideal osmocompatible substance against salt stress (Klähn and Hagemann, 2011). In a study of adaptations to salt tolerance in *Synechocystis* sp. PCC 6803, the accumulation of glucosylglycerol was much higher than sucrose, and the contribution of glucosylglycerol was much higher than that of sucrose during the salt adaptation process; therefore, in natural selection, sucrose was more critical as an energy storage substance rather than playing a role in salt tolerance (Klähn et al., 2021). As a compatible solute retained by natural evolution in halophilic bacteria (Göller et al., 1998), ectoine was believed to have unique advantages in salt tolerance. Here, to clarify the role of ectoine and exclude the influence of the native compatible solute sucrose in salt stress adaption in Syn7942, the sucrose production pathway of Syn7942 was knocked out to obtain strain Syn7942/Δsps. The growth of Syn7942/Δsps was measured under the conditions of 0, 100, 200, 300, 400, and 500 mM NaCl (Supplementary Figure S1C), and the results showed that the OD_750_ of Syn7942/Δsps on the 7th day was decreased to 62% under the 100 mM NaCl condition, 36% under the 200 mM NaCl condition compared with WT, and the strain could not survive under the 300 mM NaCl condition (Figure 3A).

Next, the ectoine synthesis module was introduced into Syn7942 instead of sucrose synthesis to obtain strain Syn7942/Δsps-ect; and the salt-adaptive ability was then investigated for growth at 0, 100, 200, 300, 400, and 500 mM NaCl, respectively (Supplementary Figure S1D). As shown in Figures 3B,C, compared with Syn7942/Δsps, the introduction of the ectoine synthesis module successfully recovered the salt tolerance of Syn7942/Δsps to WT level under 100, 200 and 300 mM NaCl conditions. Ectoine production was also determined in Syn7942/Δsps-ect grown under different salt concentrations. As shown in Figure 3D, the yield of ectoine on the 7th day was 53 mg/l under 0 mM NaCl, the similar level as that of Syn7942/NSI-ect. The yields of ectoine in Syn7942/Δsps-ect grown under 100, 200, and 300 mM NaCl conditions were 115.2 mg/L, 112.5 mg/L, and 72.9 mg/L, respectively, which were slightly improved compared with the yields in Syn7942/NSI-ect under the same conditions, probably due to that an additional part of ectoine need to be synthesized to make up for the lack of sucrose under the condition of salt stress. In addition, in Syn7942/Δsps-ect the lack of sucrose accumulation saved a part of carbon source for ectoine synthesis.

### Global transcriptomic analysis

To explore the working mechanism of ectoine in salt stress adaptation in Syn7942/Δsps-ect, comparative transcriptomic analysis was performed in Syn7942/Δsps-ect and WT, under 0 and 300 mM NaCl conditions, respectively. After data quality control, more than 16 million reads per sample were mapped to the reference genome, and the detected genes in each sample covered more than 94% of all the predicted genes (Supplementary Table S3).

**Figure 4:**
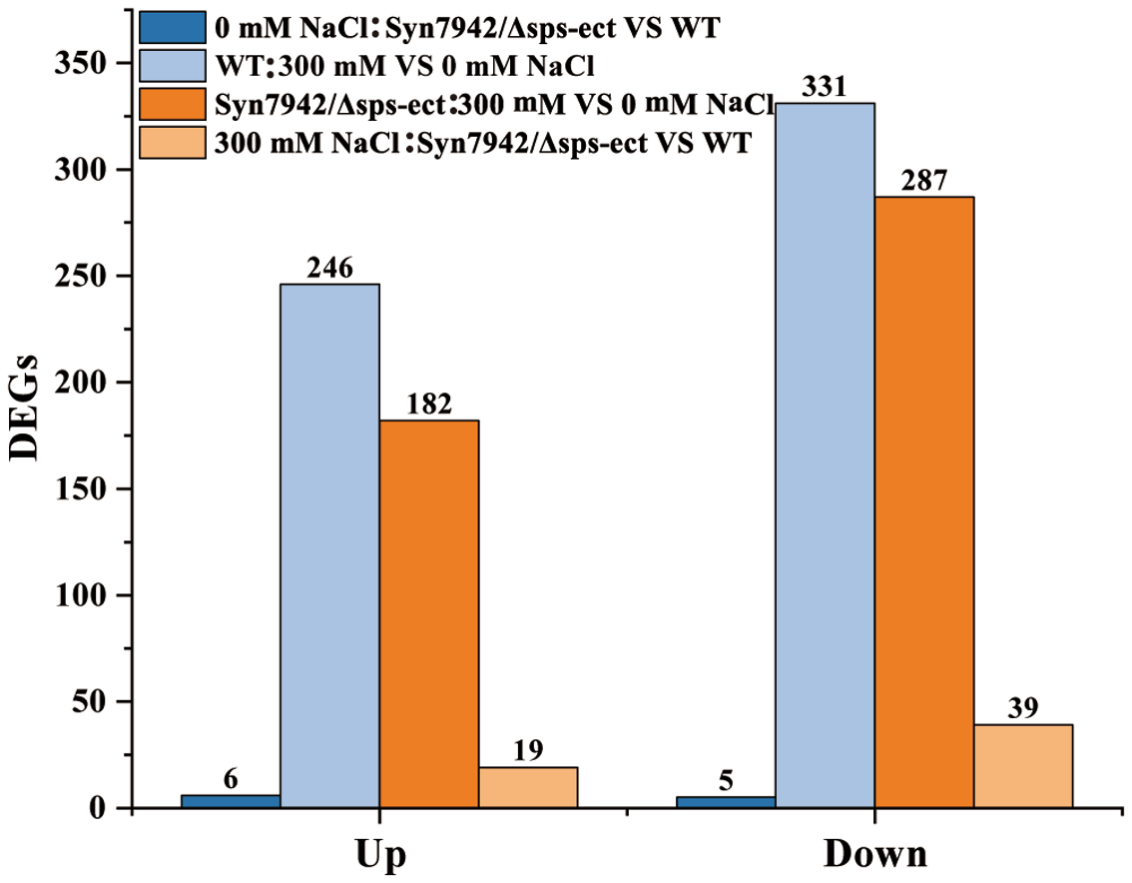
DEGs identified in transcriptomic analysis. WT: 300 mM *VS* 0 mM NaCl indicates the DEGs of WT under 300 mM NaCl compared with 0 mM NaCl condition; Syn7942/Δsps-ect: 300 mM *VS* 0 mM NaCl indicates the DEGs of Syn7942/Δsps-ect under 300 mM NaCl compared with 0 mM NaCl condition; 0 mM NaCl: Syn7942/Δsps-ect *VS* WT indicates the DEGs of Syn7942/Δsps-ect grown in 0 mM NaCl condition compared to WT; 300 mM NaCl: Syn7942/Δsps-ect *VS* WT indicates the DEGs of Syn7942/Δsps-ect grown in 300 mM NaCl condition compared to WT.

As shows in Figure 4, under 0 mM NaCl condition, only 11 DEGs were identified between Syn7942/Δsps-ect and WT. This indicated that under the condition of 0 mM NaCl, ectoine synthesis did not have significant effect on the overall metabolism.

#### Analysis of global regulation of salt adaptation in WT and Syn7942/Δsps-ect

In the WT, 246 genes were up-regulated and 331 down-regulated when grown in presence of 300 mM NaCl compared to the reference conditions (0 mM NaCl). The same comparison revealed 182 up-regulated and 287 down-regulated genes in strain Syn7942/Δsps-ect (Figure 4). Among them, 104 up-regulated and 198 down-regulated genes were shared between WT and Syn7942/Δsps-ect (Supplementary Figure S3), suggesting that these DEGs played important roles in the salt stress adaption process in both strains of Syn7942/Δsps-ect and WT.

**Figure. 5:**
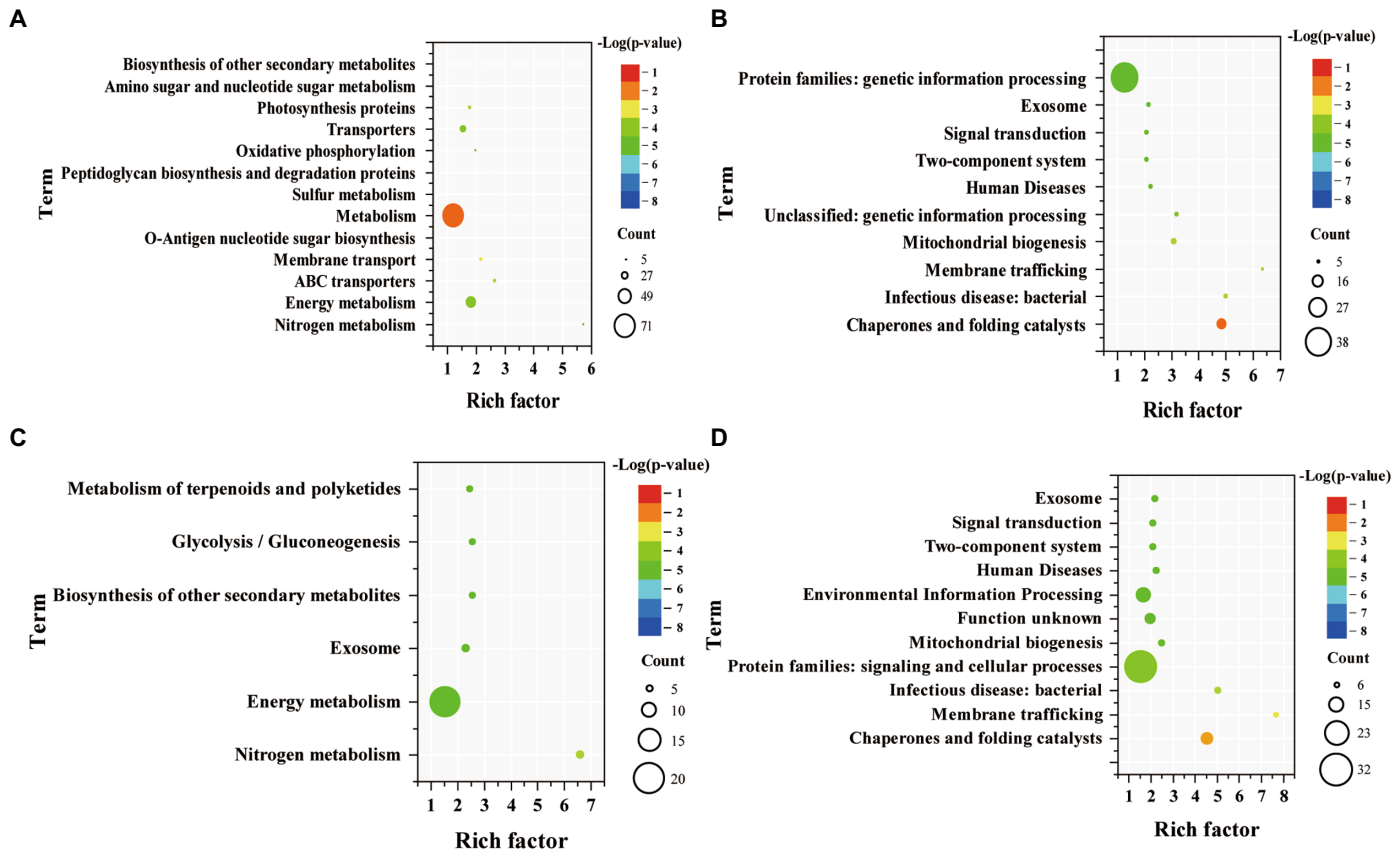
Enriched pathway terms of DEGs in WT and Syn7942/Δsps-ect. **(A)** Enriched pathway terms based on up-regulated genes of WT; **(B)** Enriched pathway terms based on down-regulated genes of WT; **(C)** Enriched pathway terms based on up-regulated genes of Syn7942/Δsps-ect; **(D)** Enriched pathway terms based on down-regulated genes of Syn7942/Δsps-ect. WT: 300 mM *VS* 0 mM NaCl indicates the DEGs of WT under 300 mM NaCl compared with 0 mM NaCl condition; Syn7942/Δsps-ect: 300 mM *VS* 0 mM NaCl indicates the DEGs of Syn7942/Δsps-ect under 300 mM NaCl compared with 0 mM NaCl condition; 0 mM NaCl: Syn7942/Δsps-ect *VS* WT indicates the DEGs of Syn7942/Δsps-ect grown in 0 mM NaCl condition compared to WT; 300 mM NaCl: Syn7942/Δsps-ect *VS* WT indicates the DEGs of Syn7942/Δsps-ect grown in 300 mM NaCl condition compared to WT.

KEGG pathway enrichment analysis was performed on DEGs of WT and Syn7942/Δsps-ect (Figure 5). Up-regulated genes of WT were mainly enriched in “nitrogen metabolism,” “energy metabolism,” “ABC transporters,” “membrane transport,” “O-antigen nucleotide sugar biosynthesis,” “metabolism,” “sulfur metabolism,” “peptidoglycan biosynthesis and degradation proteins,” “oxidative phosphorylation,” “transporters,” “photosynthesis proteins,” “amino sugar and nucleotide sugar metabolism,” and “biosynthesis of other secondary metabolites.” Compared with up-regulated genes of WT, the number of enriched pathways of up-regulated genes of Syn7942/Δsps-ect was less, mainly enriched in “nitrogen metabolism,” “energy metabolism,” “exosome,” “biosynthesis of other secondary metabolites,” “glycolysis/gluconeogenesis” and “metabolism of terpenoids and polyketides.”

Among them, “energy metabolism” and “nitrogen metabolism” were both enriched in the up-regulated genes of WT and Syn7942/Δsps-ect, but down-regulated under short-term salt stress (Billis et al., 2014). At the same time, in WT and Syn7942/Δsps-ect, genes *Synpcc7942_2016*, *Synpcc7942_2105* and *Synpcc7942_2107*, which encode nitrogen transporters, were significantly up-regulated, with an up-regulation fold of more than 8 folds. This showed that the enhancement of “energy metabolism” and “nitrogen metabolism” played important roles in helping the strains adapt to the high-salt environment for a long time. In a previous report, the operon Synpcc7942_2203 - Synpcc7942_2235 was unregulated in Syn7942 after 24 h salt stress (Billis et al., 2014). This phenomenon was not observed in this study, which may be caused by different salt stress times (in this experiment, strains were treated 4 days in 300 mM NaCl condition). The key gene of sucrose synthesis, *Synpcc7942_0808* encoding sucrose-phosphate synthase did not show significant change during salt stress (log_2_(fold-change = 0.83)). In addition, the “glycolysis/gluconeogenesis” pathway was enriched in the Syn7942/Δsps-ect. The enhancement of this pathway may facilitate ectoine production. It was reported that in the process of optimizing the biosynthesis of ectoine, increasing the carbon flux to ectoine is an important strategy (Ning et al., 2016; Giesselmann et al., 2019; Chen J. et al., 2020). The genes Synpcc7942_1312, Synpcc7942_1244 and Synpcc7942_0247 encoding ATP synthase were all up-regulated in WT and Syn7942/Δsps-ect, and the increase of ATP synthase expression was beneficial to the adaptation of cyanobacteria to high-salt environment (Soontharapirakkul et al., 2011).

The down-regulated genes of WT were mainly enriched in “chaperones and folding catalysts,” “infectious disease: bacterial,” “membrane trafficking,” “mitochondrial biogenesis,” “unclassified: genetic information processing,” “human diseases,” “two-component system,” “signal transduction,” “exosome” and “protein families: genetic information processing” (Figure 5B). Moreover, the down-regulated genes enrichment pathway analysis of Syn7942/Δsps-ect and WT were basically same (Figures 5B,D). The down-regulated genes of Syn7942/Δsps-ect were mainly enriched in “chaperones and folding catalysts,” “membrane trafficking,” “infectious disease: bacterial,” “protein families: signaling and cellular processes,” “mitochondrial biogenesis,” “function unknown,” “environmental information processing,” “human diseases,” “two-component system,” “signal transduction” and “exosome” (Figure 5D). Genes related to signal transduction were down-regulated in WT and Syn7942/Δsps-ect, which was consistent with previous reports (Billis et al., 2014). This indicated that during the long-term salt adaptation process, the contribution of the signal transduction system to the strain’s adaptation process under the high-salt environment decreased.

#### Analysis of differentially expressed genes in Syn7942/Δsps-ect compared to WT

In strain Syn7942/Δsps-ect, 19 genes were up-regulated and 39 down-regulated genes when grown in presence of 300 mM NaCl compared to the reference strains (WT). Among the DEGs, the gene *Synpcc7942_1531* encoding the molybdenum ABC transporter and the gene *Synpcc7942_1530* encoding the molybdenum-pterin binding domain were up-regulated to 4.6 and 3.9 folds, respectively. In bacteria, ingested molybdenum can combine with molybdenum cofactors, regulate the activity of molybdenum enzymes and participate in the processes of the carbon cycle, sulfur metabolism, and nitrogen fixation in life activities (Zupok et al., 2019). In addition, intracellular molybdenum homeostasis plays an important role in the process of salt environment adaptation in *Arabidopsis thaliana* (Zupok et al., 2019; Huang et al., 2021). Thus, a molybdenum-mediated salt adaptation mechanism might also contribute to the salt acclimation process, and highlighted in Syn7942/Δsps-ect. The gene *Synpcc7942_2401* encoding the heat shock protein Hsp20 was up-regulated to 2.9 folds. It was reported that the expression of *Hsp20* gene of *Oryza sativa* had a positive response to heat and high-salt environment, and the *Hsp20* gene of rice could improve the heat tolerance and salt tolerance of *E. coli* and *Pichia pastoris* (Guo et al., 2020). Therefore, *Hsp20* (*Synpcc7942_2401*) might contribute to the salt acclimation process of Syn7942.

**Figure. 6:**
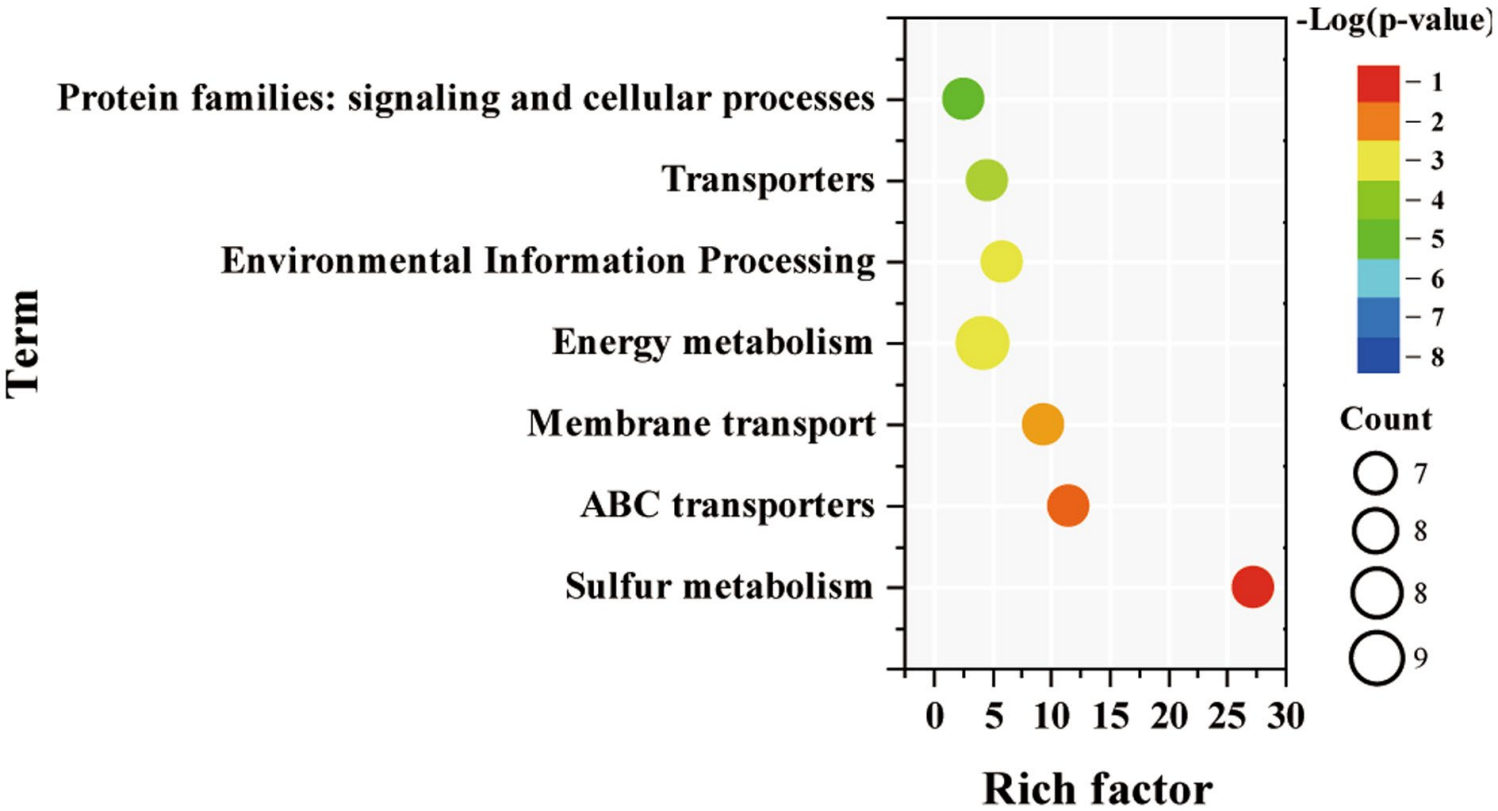
Enriched pathway terms of down-regulated DEGs in Syn7942/Δsps-ect grown in 300 mM NaCl condition compared to WT.

Among them, KEGG pathway enrichment analysis was then performed separately for the up-regulated genes of Syn7942/Δsps-ect. The results showed that down-regulated genes were mainly enriched in “sulfur metabolism,” “energy metabolism,” “ABC transporters,” “membrane transport,” “environmental information processing,” “transporters’ and “protein families: signaling and cellular processes” (Figure 6). The up-regulated genes of Syn7942/Δsps-ect was not enriched for any pathway.

Sulfur metabolism was enriched among down-regulated DEGs of Syn7942/Δsps-ect. There were 7 down-regulated genes related to sulfur metabolism, namely *Synpcc7942_1681*, *Synpcc7942_1686*, *Synpcc7942_1722*, *Synpcc7942_1682*, *Synpcc7942_1687*, *Synpcc7942_1688* encoding sulfur transporters, and *Synpcc7942_1689* encoding thiosulfate/3-mercaptopyruvate sulfurtransferase. These 6 DEGs encoding sulfur transporters were also enriched in ABC transporters. This indicated that the Syn7942/Δsps-ect might reduce sulfur requirement during salt adaptation. Sulfur metabolism plays a vital role in the process of biological stress resistance. Sulfur has been reported to have essential functions in plants’ salt adaptation, active oxygen elimination, and heat adaptation (Ali et al., 2021; Jahan et al., 2021; Zhou et al., 2022), while cyanobacteria have a similar sulfur metabolism as plants (Kharwar et al., 2021). Consistantly, sulfur metabolism-related genes were significantly up-regulated in WT and Syn7942/Δsps-ect compared to reference conditions (Figures 5A,C). Notably, the expression levels of genes related to sulfur metabolism were lower in Syn7942/Δsps-ect than in WT (Figure 6), indicating that ectoine synthesis might reduce the intensity of sulfur metabolism which was required for salt adaptation. This might be due to the protective effect of ectoine on protein (Bilstein et al., 2021), which reduced protein damage during salt adaptation.

In this study, to improve the salt tolerance of the model cyanobacterium Syn7942, the first study of heterologous synthesis of ectoine in cyanobacteria was achieved, and the salt tolerance of Syn7942 was successfully improved. Then, the recovery of salt tolerance of the Syn7942/Δsps-ect strain was achieved by introducing the ectoine synthesis pathway into the sucrose synthesis deficient strain (Syn7942/Δsps), indicating that ectoine was able to substitute for sucrose to combat against salt stress in Syn7942. After the replacement of sucrose by ectoine, the metabolic changes in the Syn7942/Δsps-ect strain was analyzed by transcriptomic analysis, which would help understand the salt adaptation mechanism of Syn7942/sps-ect. This study provided valuable information for understanding the salt tolerance mechanism of Syn7942, as well as potential candidates targets for further engineering the salt tolerance of Syn7942.

## Data availability statement

The datasets presented in this study can be found in online repositories. The names of the repository/repositories and accession number(s) can be found below: GEO GSE222067.

## Author contributions

LC, TS, and WZ: conceived and designed the study. ZD: performed the experiments. ZD, TS, WZ, and LC: analyzed the data and wrote the manuscript. All authors read and approved the manuscript.

## Funding

This research was supported by grants from the National Key Research and Development Program of China (Grant nos. 2021YFA0909700, 2020YFA0906800, 2018YFA0903600 and 2019YFA0904600).

## Conflict of interest

The authors declare that the research was conducted in the absence of any commercial or financial relationships that could be construed as a potential conflict of interest.

## Publisher’s note

All claims expressed in this article are solely those of the authors and do not necessarily represent those of their affiliated organizations, or those of the publisher, the editors and the reviewers. Any product that may be evaluated in this article, or claim that may be made by its manufacturer, is not guaranteed or endorsed by the publisher.

## Supplementary material

The Supplementary material for this article can be found online at:

https://www.frontiersin.org/articles/10.3389/fmicb.2023.1123081/full#supplementary-material

Supplementary Figure S1 Growth curves of WT, Syn7942/NSI-ect, Syn7942/Δsps, and Syn7942/Δsps-ect. **(A)** Growth curves of WT under 0, 100, 200, 300, 400, 500 mM NaCl; **(B)** Growth curves of Syn7942/NSI-ect under 0, 100, 200, 300, 400, 500 mM NaCl; **(C)** Growth curves of, Syn7942/Δsps under 0, 100, 200, 300, 400, 500 mM NaCl; **(D)** Growth curves of, Syn7942/‑sps-ect under 0, 100, 200, 300, 400, 500 mM NaCl.

Supplementary Figure S2 Standard curve of ectoine external standard method.

Supplementary Figure S3 The number of DEGs in WTand Syn7942/Δsps-ect. **(A)** The number of up-regulated genes; **(B)** The number of down-regulated genes. WT: 300 mM VS 0 mM NaCl indicates the DEGs of WT under 300 mM NaCl compared with 0 mM NaCl condition; Syn7942/Δsps-ect: 300 mM VS 0 mM NaCl indicates the DEGs of Syn7942/Δsps-ect under 300 mM NaCl compared with 0 mM NaCl condition; 0 mM NaCl: Syn7942/Δsps-ect VS WT indicates the DEGs of Syn7942/Δsps-ect grown in 0 mM NaCl condition compared to WT; 300 mM NaCl: Syn7942/Δsps-ect VS WT indicates the DEGs of Syn7942/Δsps-ect grown in 300 mM NaCl condition compared to WT.
