# Supplementary figures and images for: Improved salt tolerance of Synechococcus elongatus PCC 7942 by heterologous synthesis of compatible solute ectoine
Source: Front Microbiol. 2023 Feb 2;14:1123081. doi: 10.3389/fmicb.2023.1123081 (PMC9932913; doi:10.3389/fmicb.2023.1123081)

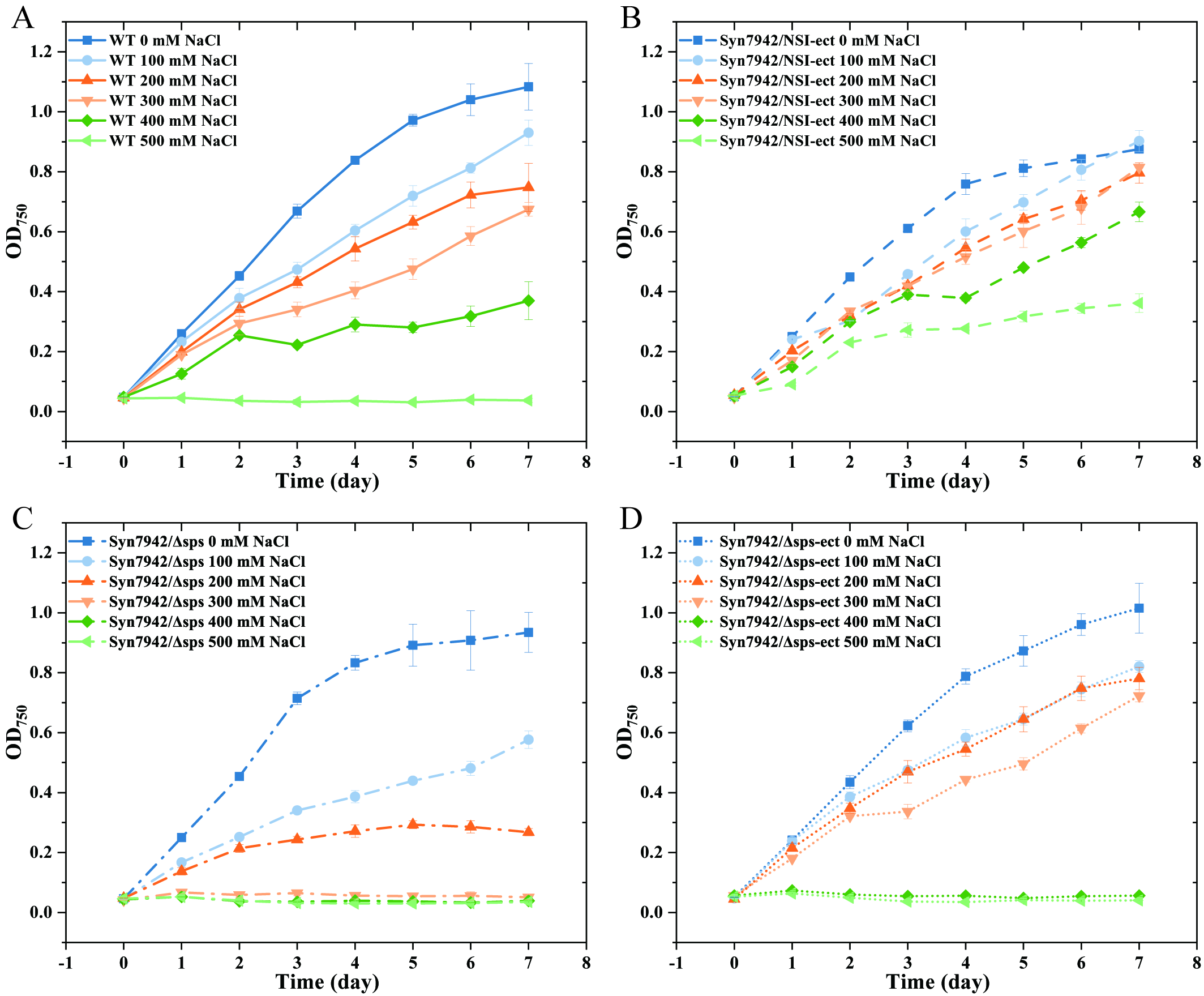

Supplement: Supplementary Figure S1 — Growth curves of WT, Syn7942/NSI-ect, Syn7942/Δsps, and Syn7942/Δsps-ect. (A) Growth curves of WT under 0, 100, 200, 300, 400, 500 mM NaCl; (B) Growth curves of Syn7942/NSI-ect under 0, 100, 200, 300, 400, 500 mM NaCl; (C) Growth curves of, Syn7942/Δsps under 0, 100, 200, 300, 400, 500 mM NaCl; (D) Growth curves of, Syn7942/‑sps-ect under 0, 100, 200, 300, 400, 500 mM NaCl. [file Image_1.TIF]

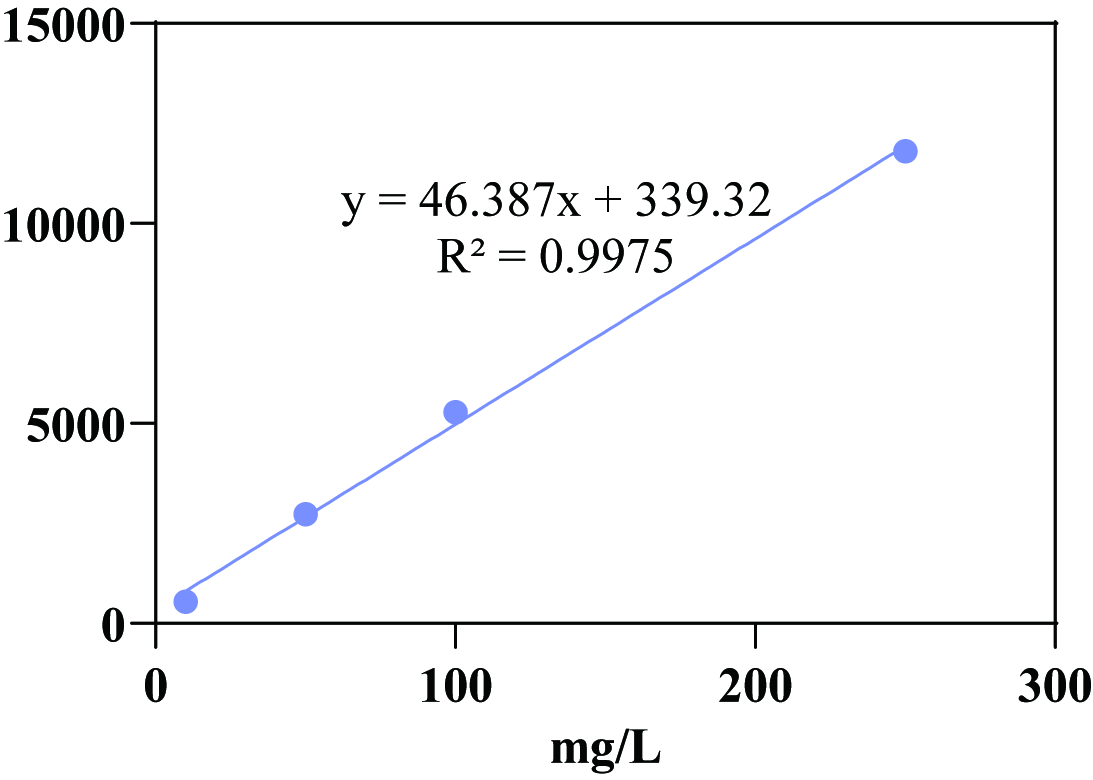

Supplement: Supplementary Figure S2 — Standard curve of ectoine external standard method. [file Image_2.TIF]

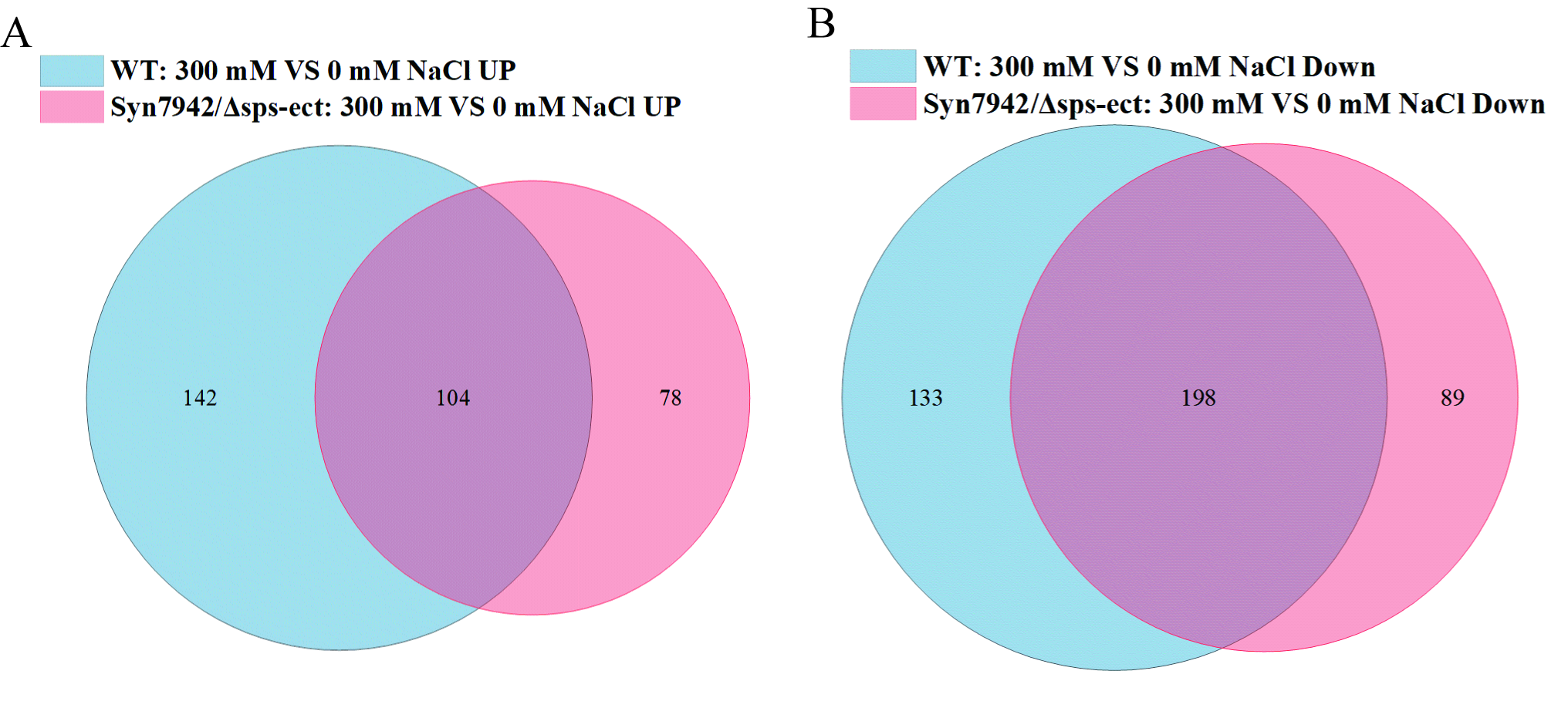

Supplement: Supplementary Figure S3 — The number of DEGs in WTand Syn7942/Δsps-ect. (A) The number of up-regulated genes; (B) The number of down-regulated genes. WT: 300 mM VS 0 mM NaCl indicates the DEGs of WT under 300 mM NaCl compared with 0 mM NaCl condition; Syn7942/Δsps-ect: 300 mM VS 0 mM NaCl indicates the DEGs of Syn7942/Δsps-ect under 300 mM NaCl compared with 0 mM NaCl condition; 0 mM NaCl: Syn7942/Δsps-ect VS WT indicates the DEGs of Syn7942/Δsps-ect grown in 0 mM NaCl condition compared to WT; 300 mM NaCl: Syn7942/Δsps-ect VS WT indicates the DEGs of Syn7942/Δsps-ect grown in 300 mM NaCl condition compared to WT. [file Image_3.TIF]
